# Impact of Fatigue on Ergonomic Risk Scores and Foot Kinetics: A Field Study Employing Inertial and In-Shoe Plantar Pressure Measurement Devices

**DOI:** 10.3390/s24041175

**Published:** 2024-02-10

**Authors:** Steven Simon, Jonas Dully, Carlo Dindorf, Eva Bartaguiz, Stephan Becker, Michael Fröhlich

**Affiliations:** Department of Sports Science, University of Kaiserslautern-Landau, 67663 Kaiserslautern, Germany; jonas.dully@rptu.de (J.D.); carlo.dindorf@rptu.de (C.D.); eva.bartaguiz@rptu.de (E.B.); stephan.becker@rptu.de (S.B.); michael.froehlich@rptu.de (M.F.)

**Keywords:** ergonomics, motion capture, observational methods, RULA, CUELA, foot pressure sensors, corporate health management

## Abstract

(1) Background: Occupational fatigue is a primary factor leading to work-related musculoskeletal disorders (WRMSDs). Kinematic and kinetic experimental studies have been able to identify indicators of WRMSD, but research addressing real-world workplace scenarios is lacking. Hence, the authors of this study aimed to assess the influence of physical strain on the Borg CR-10 body map, ergonomic risk scores, and foot pressure in a real-world setting. (2) Methods: Twenty-four participants (seventeen men and seven women) were included in this field study. Inertial measurement units (IMUs) (n = 24) and in-shoe plantar pressure measurements (n = 18) captured the workload of production and office workers at the beginning of their work shift and three hours later, working without any break. In addition to the two 12 min motion capture processes, a Borg CR-10 body map and fatigue visual analog scale (VAS) were applied twice. Kinematic and kinetic data were processed using MATLAB and SPSS software, resulting in scores representing the relative distribution of the Rapid Upper Limb Assessment (RULA) and Computer-Assisted Recording and Long-Term Analysis of Musculoskeletal Load (CUELA), and in-shoe plantar pressure. (3) Results: Significant differences were observed between the two measurement times of physical exertion and fatigue, but not for ergonomic risk scores. Contrary to the hypothesis of the authors, there were no significant differences between the in-shoe plantar pressures. Significant differences were observed between the dominant and non-dominant sides for all kinetic variables. (4) Conclusions: The posture scores of RULA and CUELA and in-shoe plantar pressure side differences were a valuable basis for adapting one-sided requirements in the work process of the workers. Traditional observational methods must be adapted more sensitively to detect kinematic deviations at work. The results of this field study enhance our knowledge about the use and benefits of sensors for ergonomic risk assessments and interventions.

## 1. Introduction

Occupational fatigue, often caused by high job demands and long duty periods, is associated with the reduced occupational health and safety of the worker, resulting in high social and financial costs [1], and is, therefore, a major problem for modern industrial societies [2,3,4]. Following the International Standardization Organization (ISO) 6385 document (2016) pertaining to the integration of ergonomics into the designs of work systems, work-induced fatigue is characterized as a non-pathological manifestation of excessive strain, either mental, local, or general, that is entirely reversible with rest [5], and diminished human performance capabilities are attributed to the inability to effectively manage physiological stressors [6]. Fatigue may impact a worker by bringing about changes in movement [7] and an immediate decline in safety-conscious work behavior, productivity, teamwork, and morale [4], and can lead to musculoskeletal disorders (MSDs) [8,9]. There may be many causes for the occurrence of fatigue at work [8], including long working hours, heavy workloads, early morning or night shifts, and insufficient sleep [10]. Furthermore, fatigue is also a problem in the context of working in a static position, like sitting for a prolonged period as in office work, and may cause a change in posture [11]. Studies have shown that unergonomic working positions correlate with increased perceived exertion [1], physical discomfort [12], and MSDs [13] that can negatively affect the work and private life quality of employees [14]. A wide body of literature describes the general causes and effects of occupational fatigue [4], while few studies have examined fatigue in the construction industry. Nevertheless, the construction industry is known as a risky and labor-intensive industry [8]. According to a meta-analysis by Hazzaa et al. [15], fatigue has the potential to alter how impact forces are absorbed.

Preventive examinations are of great importance in promoting occupational health [16,17]. Wearable devices are widespread solutions aimed at enhancing work efficiency, promoting the well-being of workers, and facilitating interactions between users and their environment at any time and location [18]. Several studies have examined kinematic or kinetic indicators to derive appropriate ergonomic measures [1,5,8,19]. Inertial measurement units (IMUs) can be employed to detect kinematic factors throughout a longer-duration work process [20,21]. Consequently, a posture score can be computed via various observational methods e.g., the Rapid Upper Limb Assessment (RULA), the Ovako Working Posture Analysis System (OWAS), Rapid Entire Body Assessment (REBA), or Computer-Assisted Recording and Long-Term Analysis of Musculoskeletal Load (CUELA) [22,23]. Vignais et al. [21] and Maurer-Grubinger et al. [20] proposed methods to use the angle distribution during work processes to calculate a final RULA score from the kinematic data.

Moreover, insole devices have been used in recent studies to detect awkward working postures and physical fatigue [8,24,25,26]. The use of in-shoe plantar pressure measurements has already been employed in studies to assess the early detection of overloading, particularly in patients with diseases such as diabetes mellitus, but also in sports [15,27] and ergonomic footwear designs [28]. As working in the construction industry involves prolonged periods of monotonous or one-sided work, it may heighten the risk of MSDs, especially foot and lower leg deformities [29]. Furthermore, the physical demands on workers may fatigue their core, foot, and lower extremity muscles [30,31] and reduce the shock absorption capacity of the foot arch [32]. Studies that have assessed the effects of flooring and shoe in-sole interventions on the level of fatigue in workers have utilized physiological, psychological, and biomechanical data collection to quantify their effects on lower back and lower extremity symptoms caused by prolonged standing [33]; however, the physical parameters that should be monitored as indicators of fatigue are still unclear [34]. Zadpoor and Nikooyan [34] have stated that past research is undecided about whether muscular fatigue leads to an increase or decrease in ground reaction forces. They highlighted two explanations. On the one hand, fatigue may diminish the capacity to adequately absorb shocks and increase the ground reaction force to counterbalance this. On the other hand, the reduction in the ground reaction force is due to the protective strategy of the human body to prevent pain or injury.

Therefore, this study aimed to assess the influence of physical strain induced through working in a real-world setting by employing the Borg CR-10 body map, ergonomic risk scores (RULA and CUELA), and in-shoe plantar pressure measurements. Furthermore, we investigated side differences that may occur because of one-sided monotonous work requirements.

## 2. Materials and Methods

Twenty-four employees in production and office (seventeen men and seven women) work settings participated in this experimental field study (age [years]: women 43.57 ± 13.56, men 37.94 ± 7.57; height [cm]: women 1.65 ± 0.05, men 1.78 ± 0.06; weight [kg]: women 72.57 ± 12.41, men 80.88 ± 12.18; body mass index [kg/m^2^]: women 26.39 ± 3.44, men 25.63 ± 3.34). Each participant was informed verbally and in writing and signed an informed consent form regarding data rights, recording videos, and participating in the study procedures. This study was conducted in accordance with the guidelines of the Declaration of Helsinki and approved by the Institutional Ethics Committee (Ethikkommission RPTU Kaiserslautern-Landau, Nr. 66).

The inclusion criteria were as follows: age > 18 years, a permanent employment contract at the company, and a minimum of 1 year of professional activity in the current professional segment. The exclusion criteria were acute restriction of physical activity in the sense of professional activity, surgical treatment of the musculoskeletal system in the last 4 weeks, and performing exhausting activities 48 h prior to the work shift, such as high-intensity weightlifting or cardio training.

### 2.1. Experimental Design

**Figure 1:**
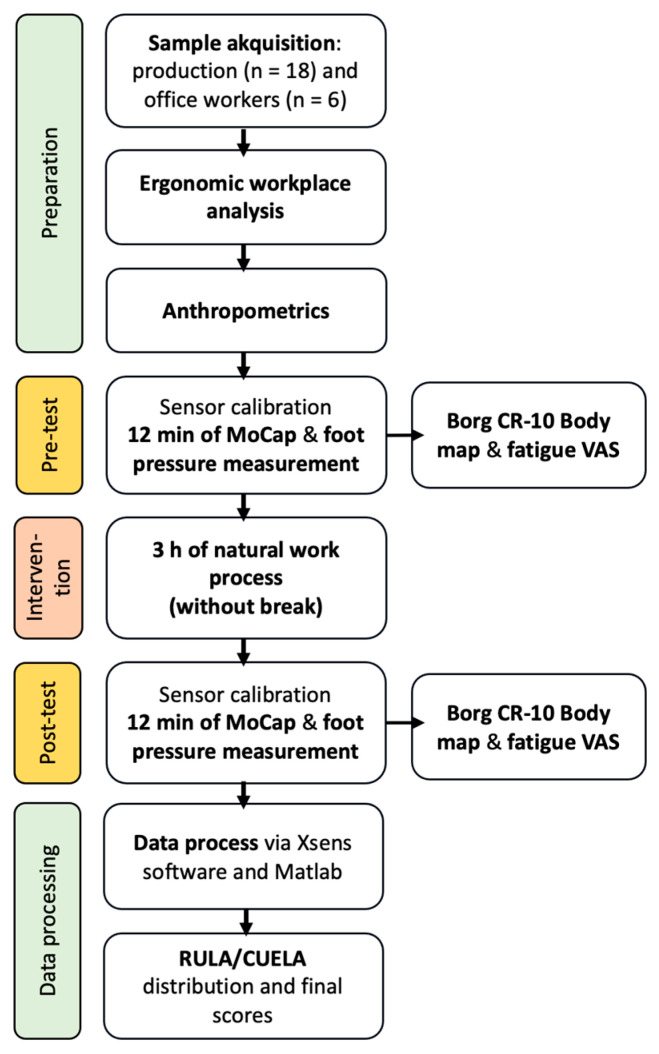
Study procedure (pre- and post-test including MoCap (n = 24) and plantar pressure measurement (n = 18)). Abbreviations: MoCap = motion capture; VAS = visual analog scale; RULA = Rapid Upper Limb Assessment; and CUELA = Computer-Assisted Recording and Long-Term Analysis of Musculoskeletal Load.

During one work shift, three participants were assessed twice by one test conductor: The subjects underwent a 12 min Motion Capture (MoCap) using IMUs (Xsens, Enschede, The Netherlands) at the start of their afternoon work shift from 2 to 10 pm (see Figure 1). After the pre-test, they worked for three hours in their common routine [17] without any breaks to monitor habitual fatigue in the specific working processes before undergoing another 12 min MoCap, in compliance with the legal requirements of German work law. The afternoon shift was selected to rule out tiredness in the sense of “sleepiness” as far as possible.

### 2.2. Experimental Tasks of the Sample

The sample included eighteen production and six office workers. The production workers engaged in tasks involving diverse physical activities. They represented several distinct workstations representing various company areas, including the manufacturing of electrical cabinets, stamping, and cabinet assembly. The office workers were recruited from the Human Resources Department, primarily performing sedentary duties for 7.5 h at desks, which involved accounting and administrative tasks requiring prolonged sitting.

### 2.3. Motion Capturing and Ergonomic Risk Score

**Figure 2:**
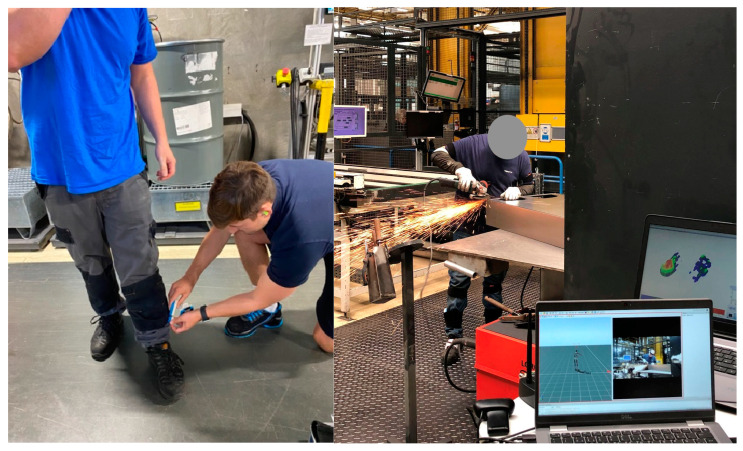
Kinematic and kinetic analysis of workers with IMUs and in-shoe plantar pressure measurement.

At the beginning of each test, the subjects’ body measurements were taken (only pre-test) and the IMUs and in-shoe pressure soles were both calibrated. Each inertial measurement unit (IMU) was comprised of a three-axis accelerometer (±16 g), a three-axis gyroscope (±2000 degrees per second), and a three-axis magnetometer (±1.9 Gauss) [35]. They represent a robust and precise reference system for reconstructing the three-dimensional motion of employees [36]. During the data collection process, while working, the sensors transmitted real-time data to a dedicated hub for synchronization (see Figure 2).

RULA assesses the risk of musculoskeletal disorders (MSDs) in workers [37]. Posture, muscle engagement, and external loads affecting body regions such as the neck, trunk, and upper limbs were evaluated using partial scores for each anatomical region (upper arm, lower arm, wrist, neck, trunk, and legs).

The RULA body part score A was determined by the muscle activity of the arms and wrists (repetition or static posture > 1 min) and forces (<2 kg, 2–10 kg, and >10 kg, repetitive or static), while the RULA body part score B was computed by the muscle activity of the neck, trunk, and legs (see above), and forces (see above). The final RULA score C depends on the A and B score values and reflects the MSD risk level. The RULA scores of participants ranged between 1 and 7. A score of 1 indicates low risk, scores of 3 or 4 indicate a necessity for intervention or procedural modifications, scores of 5 or 6 imply an impending need for alterations, and a score of 7 denotes a pressing requirement for a change in work procedures [38].

Furthermore, the Computer-Assisted Recording and Long-Term Analysis of Musculoskeletal Load (CUELA = Computer-unterstützte Erfassung und Langzeitanalyse des Muskel-Skelett-Systems according to Deutsche Gesetzliche Unfallversicherung Information 208-033) results were used for the kinematic analysis by the angle distribution in colors (part one) [39]. CUELA is a posture measurement system that aims to reproduce work activities in native work environments. The standardized assessment of physical stress is carried out for several body regions according to established occupational physiological, biomechanical, and epidemiological findings [39]. It captures real-time data during authentic work scenarios and allows study participants to execute routine tasks [40] and results in three angle categories: green (acceptable), yellow (limited acceptable), and red (unacceptable) [41].

### 2.4. In-Sole Plantar Pressure Measurement

**Figure 3:**
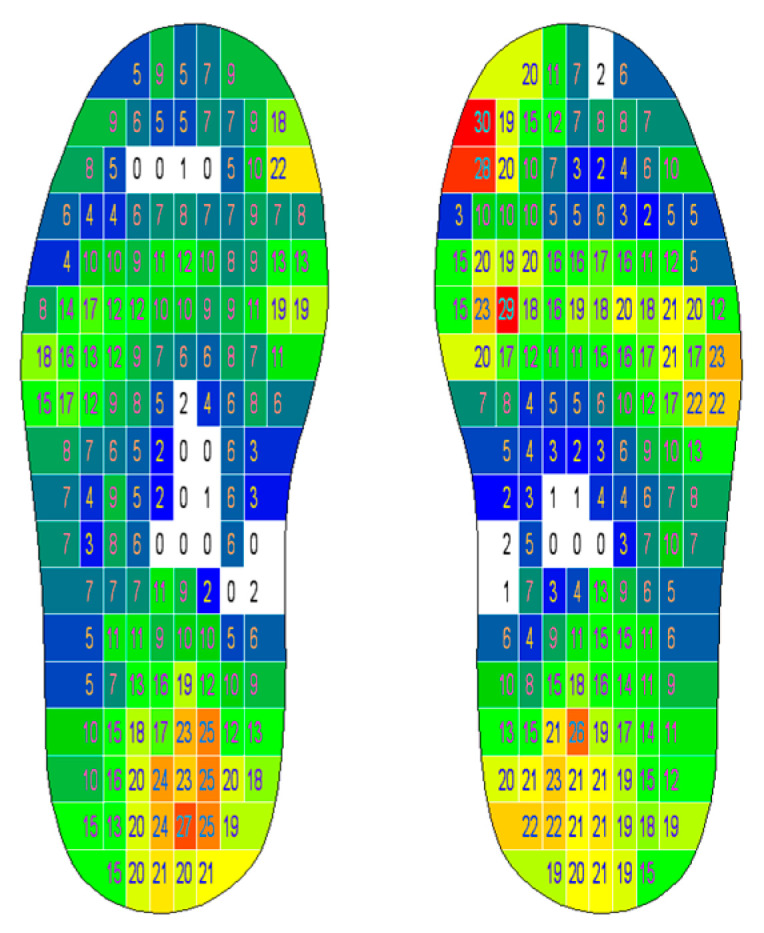
A representative image showing foot pressure measurement output (N/cm^2^) (software: T&T medilogic Medizintechnik GmbH).

Foot pressure was recorded synchronously with the MoCap system using in-shoe plantar pressure sensors in Medilogic soles (T&T Medilogic Medizintechnik GmbH, Schönefeld, Germany) [42] (see Figure 3). Each sole was equipped with a maximum of 240 surface-resistive SSR sensors depending on the assigned shoe size, with a measurement range of 0.6 to 64 N/cm^2^. The maximum measurement error was specified by the manufacturer at 5% of the full-scale output, and a sampling rate of 100 Hz was selected. The sensors operated wirelessly (WLAN) without disturbing the work process and were recalibrated before each measurement session [42].

The pressure of the foot on the insole that was caused by the lace binding of the safety shoes was eliminated by manual zeroing in a sitting position with the feet raised in a relaxed position. At this time, three to five sensors (depending on the foot size; EU 39/41/43/45) placed on the outside of the rear foot were excluded before data processing to rule out any influence of curvature in the outer shoe due to the safety shoe guidance. Each participant wore the same neutral safety shoes from the same manufacturer (Louis Steitz Secura GmbH and Co. KG) in their shoe size to exclude shoe effects [43].

### 2.5. Workers’ Physical Exertion and Fatigue

The afternoon shift was selected to rule out tiredness in the sense of “sleepiness” as far as possible. Ahsberg et al.’s [44] results showed that fatigue was highest on the night shift and lowest on the afternoon shift. The timeline of subject acquisition was randomized, and the intervention time was standardized. The interventional working time of three hours between the two measurement times followed Maman et al. [17].

After each MoCap session, participants were asked for their level of exertion (BORG CR-10 + body map) [45]. The body map has the advantage of the differentiation between different body regions. A rating on this scale is a reliable measure of the general perception of fatigue [12]. Controlled laboratory studies have concluded that there is a close relationship between work demands (the percentage of the individual physical capacity) and perceived physical exertion [46]. A cut-off value of ≥3.5 appeared to be optimal when predicting future pain, both in the neck and lower back [47]. Jakobsen et al. [48] used this scale to evaluate the fatigue levels in 200 workers and summarized that an exertion level of at least 4 can be an indicator of high muscular load. Cruz-Montecinos et al. demonstrated that physical exertion can be a good predictor of neuromuscular fatigue [49]. Additionally, the perceived level of fatigue was measured using a 10 mm visual analog scale (VAS), allowing for real-time detection [50].

### 2.6. Data Processing

**Figure 4:**
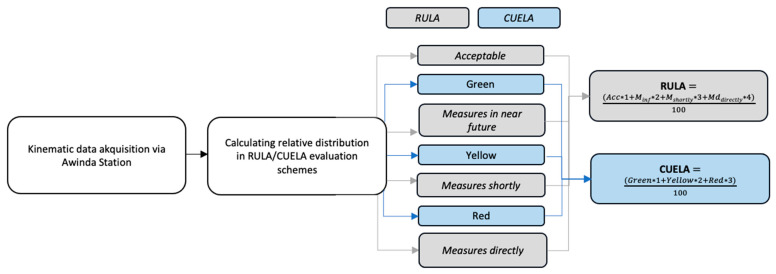
Workflow for evaluating final RULA and CUELA scores over two 12 min working periods (M_1_ and M_2_).

The Borg body map exertion values were used to compute a final score, an upper extremity score (UE-score), a lower extremity score (LE-score), and a core score. The relevant ergonomic kinematic variables were rated based on the RULA score using a self-written MATLAB script (MathWorks, Natick, Massachusetts, United States). The working time in four levels was calculated over the whole working process: *acceptable*, *measures should be initiated in the near future*, *measures should be initiated shortly,* and *measures should be initiated directly*. Finally, the distribution was transferred to a final RULA score that represented the dynamics of the whole working process. For CUELA, the relative distribution in *green*, *yellow*, and *red* was calculated and used to assess a final score to make it comparable to RULA (see Figure 4).

After the data assessment, the vertical ground reaction forces of the foot pressure measurement insoles were exported and divided into six different foot regions that were adapted from a previous study [51]:

- Rearfoot (0–30% length; 0–100% width);
- Midfoot (31–60% length; 0–100% width);
- Metatarsal heads (61–80% length; 0–100% width);
- Forefoot (81–100% length; 0–100% width);
- Inner foot (0–100% length; 0–60% width);
- Outer foot (0–100% length; 61–100% width).

To evaluate the raw values of the pressure measurement sensors of the right and left insoles, all measurements first had to be exported to CSV files. Using MATLAB software, the individual measurements were merged and sorted in tabular form according to the specified variables for the pre- and post-tests (see Section 3). The raw data from the pressure insoles from each worker were used to calculate mean values and the percentage of time this area was loaded using MATLAB. Additionally, the load times of the individual sensors of the foot areas were used to calculate mean values to eliminate time effects and differences in foot areas. Furthermore, for each worker, the mean peak pressure from the sensors in each foot area was calculated as well as the impulse values [43,52,53]. In contrast with Caravaggi et al. [52], rather than obtaining the highest value from a range, the individual maximum values from each sensor were used to compute the mean values. The (pressure) impulse was calculated from the product of pressure [N/cm^2^] and time [s] for each sensor. As a result, areas where a mean load prevailed over a longer period were highlighted. While short-term high loads were particularly apparent in the peak pressure evaluation, the impulse evaluation emphasized areas where a medium load prevailed over a longer period.

### 2.7. Data Analysis

First, all data were checked for normal distributions by the Shapiro–Wilk test via SPSS software (IBM, version 29, SPSS Inc., Chicago, IL, USA), and a visual analysis of the data was conducted to detect outliers. The differences between the pre- and post-fatigue levels were normally distributed, as assessed by the Shapiro–Wilk test; however, this was not the case for the Borg body map UE- and LE-scores, RULA scores, and CUELA pre-test results. Simulation studies have shown that repeated-measures ANOVA (rmANOVA) is a robust test for violations of the normal distribution assumption when it is the only assumption that is violated [54]. Adjusted p-values, determined with rmANOVA, were compared with the alpha level of 0.05, and the effect size following Cohen [55] was assessed. For the means, peak pressures, impulse values, and load times in the six areas of the foot, rmANOVAs were used. We decided to not exclude any outliers. Post hoc analysis was conducted with the Bonferroni adjustment. Calculations and visualizations were performed in MATLAB, SPSS, and the Python library “Seaborn” software packages [56].

## 3. Results

### 3.1. Physical Exertion and Fatigue

**Figure 5:**
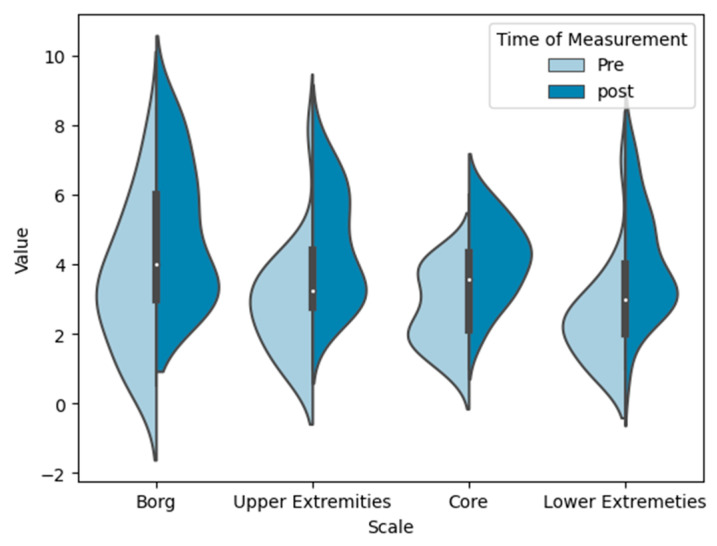
Violin plots representing the Borg CR-10 body map of the sample (n = 24) as total (pre-test: median—3, interquartile range (IQR)—2.78; post-test: median—4.50, IQR—3.13), and for the UEs (upper extremities) (pre-test: median—2.88, IQR—1.75; post-test: median—4.19, IQR—2.72), core (pre-test: median—2.88, IQR—1.75; post-test: median—4.22, IQR—1.81), and LEs (lower extremities) (pre-test: median—2.17, IQR—1.25; post-test: median—3.17, IQR—2.08). The violin shape was created by plotting a kernel density estimate of the data.

Figure 5 presents the Borg CR-10 body map results for each body segment (UE, LE, and core). Physical exertion in the post-test (4.96 ± 1.96) was shown to be higher than in the pre-test (3.49 ± 2.01). The rmANOVA with a Greenhouse–Geisser correction determined that mean exertion levels showed a highly statistically significant difference between measurements (F(1, 23) = 27.751; *p* < 0.001; partial η^2^ = 0.55). The Bonferroni-adjusted post hoc analysis significantly confirmed (*p* < 0.001) higher exertion scores after a 3 h intervention (Mean_Diff_ = 1.54; 95% confidence interval [CI] = 0.93–2.14).

**Figure 6:**
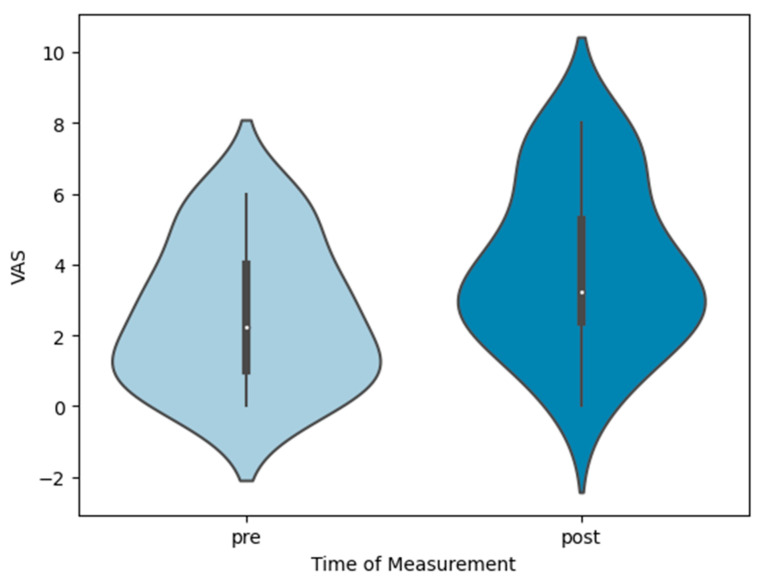
Violin plots show fatigue visual analog scale of the sample (n = 24) regarding pre-test (median: 2.25; IQR: 3) and fatigue VAS post-test (median: 3.25; IQR: 2.88).

Fatigue levels also had a highly significant measurement difference (see Figure 6) (F(1, 23) = 13.979; *p* < 0.001; η_p_^2^ = 0.38). The post hoc analysis (Bonferroni correction) revealed a significant difference between the pre- and post-tests (Mean_Diff_ = 1.39; 95% CI = 0.62–2.16).

### 3.2. Ergonomic Risk Scores

Both RULA and CUELA scores were slightly lower in the post-test than in the pre-test (see Table 1), but the statistical analysis (rmANOVA) revealed no significant difference between the measurements (F(1, 23) = 2.62; *p* = 0.12).

### 3.3. In-Sole Plantar Pressure Measurement

The percentage of loaded sensors among the total number of sensors did not significantly change across both sides and measurement times (see Table 2).

**Figure 7:**
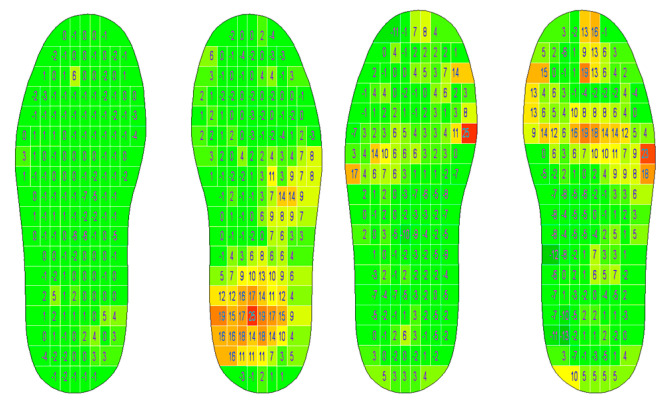
Differences between mean pressures (positive values = post-test > pre-test; negative values = post-test < pre-test) in subjects 2 (**left**) and 5 (**right**) (N/cm^2^) (software: T&T medilogic Medizintechnik GmbH).

The differences between the pre- and post-tests were calculated (Figure 7). The rmANOVA showed no statistically significant difference in the mean plantar pressure values between measurements (F(1, 17) = 0.176; *p* = 0.68) but did show significant differences in the mean pressures between the foot sides (F(1, 17) = 22.402; *p* < 0.001; η_p_^2^ = 0.57). Bonferroni-adjusted post hoc analysis confirmed significantly (*p* < 0.001) higher plantar pressure values in the right foot than in the left foot (Mean_Diff_ = 230.693; 95% CI = 201.702–259.685).

**Figure 8:**
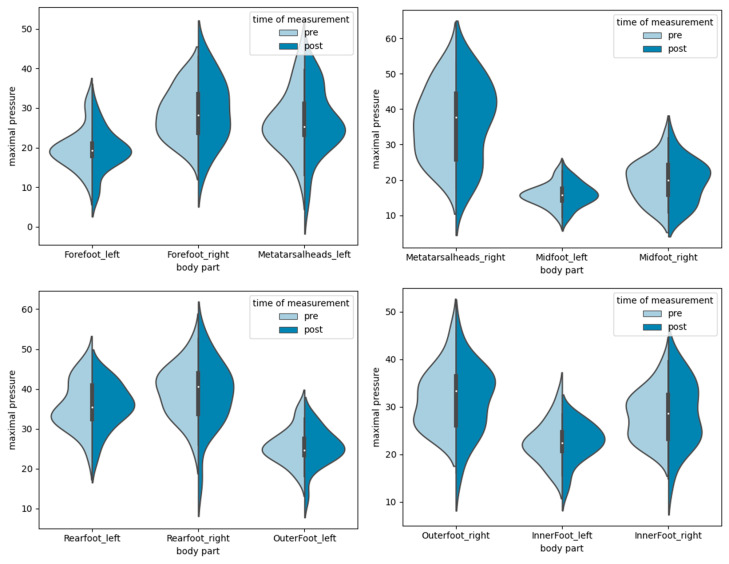
Pre- and post-test results of peak pressures in six areas of the foot for left and right sides (N/cm^2^) of production workers (n = 18): forefoot (median: 24.12; IQR: 6.14), metatarsal heads (median: 31.34; IQR: 12.51), midfoot (median: 17.65; IQR: 5.87), rearfoot (median: 37.66; IQR: 9), outer foot (median: 28.77; IQR: 6.69), and inner foot (median: 25.53; IQR: 6.59).

In line with the mean pressure values, the peak pressures showed no significant differences between the pre- and post-tests (F(1, 17) = 0.794; *p* = 0.385) but did show significant side differences between the left and right foot peak pressures (F(1, 17) = 19.84; *p* < 0.001; η_p_^2^ = 0.54), confirmed by post hoc analysis (Mean_Diff_ = 5.34; 95% CI = 2.81–7.869). The time load in the six areas of the foot showed no significant differences between the pre- and post-tests (F(1, 17) = 0.911; *p* = 0.353). The descriptive data of peak pressures are presented in Figure 8.

Regarding impulse values (Ns/cm^2^), the data showed an increase between the time of measurements, especially in the metatarsal heads (see Table 3), but the statistical analysis with rmANOVA revealed no significant difference between measurements (F(1, 17) = 1.07; *p* = 0.317) apart from between the sides (F(1, 17) = 5.85; *p* = 0.027). The post hoc analysis confirmed the presence of significant side differences (Mean_Diff_ = 4.67; 95% CI = 0.60–8.74).

## 4. Discussion

### 4.1. Results

The statistical analysis showed that physical exertion (pre: 3.26 ± 1.82; post: 5.09 ± 1.92) and fatigue (pre: 2.58 ± 1.97; post: 3.97 ± 2.29) were significantly increased. Regarding the Borg body map data, levels between two and four represent moderate exertion, and levels over four represent high exertion. A fatigue level between two to five stands for mild fatigue. This implies that an increase from moderate to high in terms of physical exertion and an increase within the mild fatigue range was detected [48,50]. RULA scores that indicate risks of 4.96 ± 0.99 (pre-test) and 4.79 ± 1.02 (post-test) correspond to levels at which further intervention is needed and changes are required [37]. Comparably, CUELA showed scores of 1.63 ± 0.16 (pre-test) and 1.57 ± 0.12 (post-test), which means that the physiological range of motion was limited but acceptable. These results can be mainly attributed to many upper body rotations, awkward lifting movements, and static or one-sided workloads, and, consequently, represent a strong basis for initiating physical and environmental interventions for both production and office workers. The results regarding work-related musculoskeletal strains were the basis for targeted preventive measures according to the “(S)TOP principle“, including substitution (avoiding highly stressful activities), technical measures (ergonomic workspace designs), organizational measures (ergonomic work organization designs), and personal measures (behavioral prevention) [39]. A practical approach was to alternate workstations that allow work processes in two directions and to make a change of activity after each shift hour. Behavioral prevention workshops were initiated to educate workers on the importance of everyday postures and lifting/pushing movements.

Despite high exertion in the post-test, the perceived level of physical fatigue in workers was still at a mild level. The observational RULA and CUELA ergonomic scores did not show significant deviations from the pre- to post-test. The fact that the authors chose the same work task in both the pre- and post-tests, and that a 2D video analysis monitored the execution of the work process at both points in time, ensured comparability. This raises questions regarding whether and to what extent these scores can help to recognize changes in physical exertion or fatigue at an individual level at an early stage in the work schedule. The level of exertion and increasing fatigue after several hours of work may not directly lead to a change in the ergonomic risk scores. A plausible explanation for the lack of an increase in the observational method scores could be that the employees, at the beginning of the work shift, have to get used to coordinative processes, while later in the work schedule, they work with more economical movements. The chosen observational methods (RULA and CUELA) may not be sensitive enough to account for small changes in the determination of individual and total scores. This highlights a need to adapt these scores more closely and precisely to the increased technical possibilities for data collection and evaluation and to address the additional informational value. Maman et al. [17] developed a data-driven approach to detect physical fatigue via four IMUs and found that the wrist, torso, and hip sensors had the strongest contribution to detecting fatigue. In this field study, 17 sensors were used, and upper extremity parameters were highlighted, as RULA is more upper-body oriented. No significant effects could be detected during the afternoon work shift of this study’s participants.

Furthermore, the results of this approach did not identify any significant differences in plantar pressure values (mean, peak, and impulse pressures) between measurements at different work shift times. The authors hypothesized that the duration of the work shifts in a standing and walking position with simultaneous high postural risk, as shown by the observational methods, and prolonged loading of the foot may change the plantar pressure values and distribution due to muscle fatigue. For this reason, only the production workers were involved in this examination. The participants had to work 3 h before post-test measurements were recorded. The pressure impulse value descriptive data show a trend toward elevated values that was not confirmed by statistical analysis. In contrast with mean and peak pressures, impulse values represent the duration of the load. While short-term high loads are particularly apparent in the peak pressure evaluation, the impulse evaluation emphasizes areas where a medium load prevails over a longer period. Recent studies have demonstrated the value of detecting fatigue by in-shoe plantar pressure sensors [8,24]. Antwi-Afari et al. [8] indicated that fatigue patterns, derived from acceleration and plantar pressure data recorded by a wearable insole device, may effectively discern physical fatigue in construction workers. Garcia et al. [30] showed that lower extremity muscle fatigue was highly evident after 5 h of standing work. The assumption regarding a change in the pressure distribution in various areas of the foot could not be confirmed. Generally, it must be noted that the study situation was weak regarding the effects of physical exertion and fatigue in the course of work shifts on the plantar vertical ground reaction forces in safety shoes. Messing et al. [57] suggested that foot pain at work may emerge from the prolonged loading of the connective tissues during prolonged standing with uncomfortable shoes and hard floors, stretching the relevant ligaments that support the longitudinal and transversal arches. Studies in the field of running have also revealed different findings in this respect. Although Hazzaa et al. [15] concluded that local muscle fatigue in running indicates a non-significant reduction in peak pressure, they described several studies that have demonstrated elevations in peak pressures and impulses in the area of the metatarsal heads, and the entire arch, especially the medial arch [58,59,60]. Headlee et al. [32] highlighted that the fatigue of foot muscles can result in a navicular drop, a lowering of the medial longitudinal arch.

Nevertheless, the kinetic analysis helped to detect ergonomically relevant side differences between the dominant- and non-dominant sides, caused by the need for one-sided work activities and/or the worker’s choice to use only the dominant side. The 2D video analysis that was used to analyze the work processes independently of the sensor measurements revealed that there were obvious one-sided requirements in the work process, but the worker had the choice to increasingly use the dominant side as the supporting and gripping side within these individual work steps. These factors might lead to the side differences highlighted by the statistical analysis. Among studies on the biomechanics of running, Brown et al. [61] found no interaction between fatigue and limb dominance when examining joint kinematics or kinetics, but Hazzaa et al. [15] observed that fatigue may accentuate the kinematic and kinetic differences between limbs. These findings may translate into a strong ergonomic benefit and were the basis for further behavioral and environmental interventions such as training for postural awareness and adapting to work conditions, work processes, and work materials.

### 4.2. Methods

The authors decided to conduct a field study representing real-world work processes since laboratory scenarios are deficient in representing actual workplace scenarios. The study participants were instructed to perform the same task (always depending on their individual workstation, for example, the manufacturing of electrical cabinets, stamping, cabinet assembly, and administrational computer work) at both measurement time slots but were not restricted in their behavior. This approach was deliberately chosen to reflect the real requirements of day-to-day work, which is seen as a major strength of this study design.

*Work-related physical exertion and fatigue detection*

The Borg CR-10 scale for discomfort is feasible for use by ergonomists and occupational healthcare providers. Even if the methodology is based on established scores, it is still a subjective survey that depends on perceptions [62]. Balogh et al. [63] emphasized that employees with musculoskeletal complaints overestimate the physical strain. The methodology relied on these subjective assessments and therefore lacked objective parameters, such as heart rate, blood lactate level, and muscle activity (surface electromyography). The absence of these critical measurements might lead to an incomplete understanding of fatigue processes, and this must be taken into account when interpreting the results. However, it may not be possible to fully identify fatigue processes even with the inclusion of these methods. We therefore opted for an economical, scientifically established methodology that is suitable and practicable in an ergonomic context.

*Kinematic analysis*

The use of sensors to detect worker exertion is increasingly becoming the focus of occupational science [64]. RULA and CUELA, which have been established assessment tools for decades in the ergonomic evaluation of work processes, were chosen to operationalize the MoCap data. Both observational methods were used to enable a comparison between different scores to improve the validity of the results. The MoCap data acquired along with the IMUs made it possible to record the kinematic data over 24 min of the work process, thus making it possible to determine the RULA score for each posture of the work process and to allow a temporal distribution in the four risk areas that represent the overall process. This may be seen as a significant enhancement of the test results; however, it was a complex data processing procedure that needed to be carried out by the research team. These computations encompassed the overall RULA scores of both body halves. Consequently, a more objective determination of the total ergonomic load was feasible, leading to a more accurate assessment of workplace ergonomics [20]. Algorithms that use IMU data to provide score-based results are an option, and proposals have recently been published [35]. Software has also been developed that allows the risk profiles of workers to be assessed in real time [19,21] based on *Deutsche Gesetzliche Unfallversicherung* (DGUV) assessment scales such as CUELA [65].

*Kinetic analysis*

As a general limitation, in-shoe plantar pressure measurements cannot measure shear forces, which may be an important factor in shifting loads and the resulting soft tissue stress on the feet. The foot pressure measurement system can record the vertical force of the ground reaction force data to evaluate the physical intensity and ergonomic risk level. Nevertheless, it is easy to use, has great potential in dynamic and complex settings, and is easily inserted in safety boots [8,28]. It does not influence movement. The whole work process, implying multiple footsteps of workers, can be measured in real-time [24]. Every participant wore the same neutral safety shoes from the same manufacturer to neutralize disturbing effects [43,52]. Bisiaux et al. [66] chose the peak pressure and relative impulse on different foot areas as ground reaction parameters for assessing fatigue. In contrast with Karvekar et al. [67], we did not include parameters such as gait speed, acceleration, stride frequency, stride length, and step width in the evaluation of the data, but these should be added in future studies.

### 4.3. Strengths and Limitations of This Study

The authors decided on a field assessment, in which great strength was observed in the representation of actual work processes and combined two different types of sensors: inertial measurement units for detecting the postural risk during two work processes at different work shift times, and plantar pressure sensors. Most studies focus on data collection in laboratory settings by using markers that make their application in construction sites very challenging [8] and the results cannot be applied to real-world settings. The combination of kinematic data with kinetic data allows statements to be made not only about postural risk but also about the vertical ground reaction forces acting on the worker’s body and thus strengthens the basis for targeted workplace interventions that can reduce the risk of developing musculoskeletal complaints in the long term. Making in-shoe plantar pressure measurements via insoles may be well-suited to identifying side differences in ergonomic settings and improving interventions. The side differences may be attributed to the one-sided work requirements, as well as to the individual behavior of the workers concerning their dominant side.

The study design included two established ergonomic scores to determine the postural risk in the work process to strengthen the informative value of the postural risk and to compare the two evaluation systems. Since CUELA, in contrast with RULA, is based on distributions in three risk areas rather than a final score, the authors made a proposal for the comparability of the two scores in the methodology, which can ensure better comparability. Refinements such as these have the potential to enhance the accuracy of assessment and provide a stronger foundation for enhancing ergonomic conditions in real-world work settings. Regarding CUELA, the angle ranges were categorized without considering external circumstances (e.g., supported upper body or arm posture). In parts 2 and 3, the score offers an evaluation of moments and forces, which could not be fulfilled within the framework of the study design.

As a primary limitation of this study, the small group size, with 18 production and 6 office workers, and the exclusion of left-handed participants must be noted, which may skew the generalizability of the results. It would be worthwhile to study whether left-handedness would change the load profile of the right dominant side, as many work processes were performed via the upper extremity.

### 4.4. Future Work

The need for accurate full-body ergonomic risk assessment in industries combining kinematic and kinetic measures persists. Methodologies that are rapid and easy to implement for ergonomists are needed, and this implies that the detection of physical fatigue and overload must be feasible, accurate, and reliable.

Regarding side differences in foot pressure, the study cohort consisted exclusively of participants with right-handed and right-sided dominant legs. This should be varied in future investigations, with an equal number of left- and right-sided participants. A combination of these parameters and more objective measurements, such as heart rate and voluntary muscle contractions, are recommended. For instance, studies in industrial surroundings have underlined the rising demand for multiple physiological metrics, such as heart rate and heart rate variability, or skin temperature, to determine fatigue [5] that were not highlighted in this study. Integrating objective measures of fatigue with current subjective methods could enhance the accuracy and applicability of ergonomic assessments in occupational settings. Future studies should examine real-world industrial tasks over a longer period (in compliance with the law) with a greater sample size and a balance between the sexes as well as right- and left-handed participants.

Furthermore, new measurement principles with intelligent systems, which proved to be applicable in other fields of study [26,68], may be used to monitor the pressure during industrial work. Including posture measurement in intelligent insole pressure capturing could be a major improvement in ergonomic research.

## 5. Conclusions

The present study showed significant differences between the pre- and post-test scores for perceived physical exertion and fatigue; however, these differences did not affect the RULA and CUELA scores. Lateral differences between the dominant and nondominant sides were identified for all pressure variables; consequently, ergonomic and educational interventions were initiated. The combination of kinematic and kinetic sensors is extremely promising for the worker and can be easily integrated into ergonomic settings. Developing more sensitive and comprehensive methods to accurately identify and address work-related physical exertion and fatigue is essential. A comprehensive understanding based on a combination of kinematic and kinetic factors will improve the intervention programs that align with the specific demands of the workers’ jobs.

## Figures and Tables

**Table 1:** Descriptive data of final ergonomic risk RULA and CUELA scores (determined via the distributions in the risk areas) of the sample (n = 24).

	RULA		CUELA	
	*Pre*	*Post*	*Pre*	*Post*
**Mean**	4.96	4.79	1.63	1.57
**Standard deviation**	0.99	1.02	0.16	0.12

**Table 2:** Percentage of loaded sensors among the total number of sensors (%) (n = 18 production workers).

	Pre		Post	
	*Left*	*Right*	*Left*	*Right*
**Mean**	86.29	88.73	84.27	87.52
**Standard deviation**	24.63	25.63	24.43	25.41

**Table 3:** Means values of impulse (pressure) in production workers (n = 18) for each foot region in the pre- and post-test comparison (Ns/cm^2^). ToM = time of measurement.

Impulse (Ns/cm^2^)	Pre-Test	Post-Test	Difference Pre–Post (+: Post-Test > Pre-Test; − = opp.)
Left metatarsal heads	7.78 ± 5.36	10.16 ± 6.88	2.39
Right metatarsal heads	13.48 ± 6.31	15.45 ± 8.49	1.97
Left midfoot	9.57 ± 3.46	10.56 ± 6.25	0.99
Right midfoot	12.75 ± 7.02	12.05 ± 6.90	−0.70
Left rearfoot (pre)	19.69 ± 7.96	21.33 ± 8.17	1.64
Right rearfoot (pre)	20.30 ± 7.09	20.78 ± 9.34	0.48
**Repeated-measures ANOVA (ToM)**			**F(1, 214) = 1.68; *p* = 0.196**

## Data Availability

Data are contained within the article.
